# A Novel Risk Score to Predict In-Hospital Mortality in Patients With Acute Myocardial Infarction: Results From a Prospective Observational Cohort

**DOI:** 10.3389/fcvm.2022.840485

**Published:** 2022-04-07

**Authors:** Lulu Li, Xiling Zhang, Yini Wang, Xi Yu, Haibo Jia, Jingbo Hou, Chunjie Li, Wenjuan Zhang, Wei Yang, Bin Liu, Lixin Lu, Ning Tan, Bo Yu, Kang Li

**Affiliations:** ^1^Department of Biostatistics, School of Public Health, Harbin Medical University, Harbin, China; ^2^Department of Cardiology, Second Affiliated Hospital of Harbin Medical University, Harbin, China; ^3^Department of Emergency, Tianjin Chest Hospital, Tianjin, China; ^4^Department of Cardiology, Tianjin Medical University General Hospital, Tianjin, China; ^5^Department of Cardiology, Fourth Affiliated Hospital of Harbin Medical University, Harbin, China; ^6^Department of Cardiology, The Second Hospital of Jilin University, Changchun, China; ^7^Department of Cardiology, Daqing Long Nan Hospital, Daqing, China; ^8^Department of Cardiology, Guangdong General Hospital, Guangzhou, China

**Keywords:** acute myocardial infarction, in-hospital mortality, risk score, logistic regression model, net reclassification improvement, integrated discrimination index

## Abstract

**Objectives:**

The aim of this study was to develop and validate a novel risk score to predict in-hospital mortality in patients with acute myocardial infarction (AMI) using the Heart Failure after Acute Myocardial Infarction with Optimal Treatment (HAMIOT) cohort in China.

**Methods:**

The HAMIOT cohort was a multicenter, prospective, observational cohort of consecutive patients with AMI in China. All participants were enrolled between December 2017 and December 2019. The cohort was randomly assigned (at a proportion of 7:3) to the training and validation cohorts. Logistic regression model was used to develop and validate a predictive model of in-hospital mortality. The performance of discrimination and calibration was evaluated using the Harrell’s c-statistic and the Hosmer-Lemeshow goodness-of-fit test, respectively. The new simplified risk score was validated in an external cohort that included independent patients with AMI between October 2019 and March 2021.

**Results:**

A total of 12,179 patients with AMI participated in the HAMIOT cohort, and 136 patients were excluded. In-hospital mortality was 166 (1.38%). Ten predictors were found to be independently associated with in-hospital mortality: age, sex, history of percutaneous coronary intervention (PCI), history of stroke, presentation with ST-segment elevation, heart rate, systolic blood pressure, initial serum creatinine level, initial N-terminal pro-B-type natriuretic peptide level, and PCI treatment. The c-statistic of the novel simplified HAMIOT risk score was 0.88, with good calibration (Hosmer–Lemeshow test: *P* = 0.35). Compared with the Global Registry of Acute Coronary Events risk score, the HAMIOT score had better discrimination ability in the training (0.88 vs. 0.81) and validation (0.82 vs. 0.72) cohorts. The total simplified HAMIOT risk score ranged from 0 to 121. The observed mortality in the HAMIOT cohort increased across different risk groups, with 0.35% in the low risk group (score ≤ 50), 3.09% in the intermediate risk group (50 < score ≤ 74), and 14.29% in the high risk group (score > 74).

**Conclusion:**

The novel HAMIOT risk score could predict in-hospital mortality and be a valid tool for prospective risk stratification of patients with AMI.

**Clinical Trial Registration:**

[https://clinicaltrials.gov], Identifier: [NCT03297164].

## Introduction

Patients with acute myocardial infarction (AMI) have a wide range of risks for immediate and long-term mortality worldwide. The in-hospital mortality of patients with AMI has decreased because of improved therapies over the past decades, such as early reperfusion, primary percutaneous coronary intervention (PCI), antithrombotic medication, and secondary prevention. However, the mortality rate of patients with AMI in China continues to substantial increase, at approximately 60 per 100,000 population annually (1). Thus, a potential risk stratification tool provides an opportunity to identify high risk patients and those who will benefit from appropriate decision-making on treatment strategy, level of care or length of hospital stay.

Over the last two decades, several risk scores have been developed to predict in-hospital mortality in patients with acute coronary syndrome (ACS) or AMI (2–12). Among them, the Global Registry of Acute Coronary Events (GRACE) (2) risk score is the most popular and widely recommended model for risk assessment and adjustment in patients with ACS/AMI in the guidelines of the European Society of Cardiology (ESC) (13, 14) and American College of Cardiology/American Heart Association (ACC/AHA) (15). Several other risk score models, such as the Acute Coronary Treatment and Intervention Outcomes Network (ACTION) Registry–Get With The Guidelines (GWTG) mortality risk score (4, 6) from the United States, the China Acute Myocardial Infarction (CAMI) registry (8, 9) or the Improving Care for Cardiovascular Disease in China-Acute Coronary Syndrome (CCC-ACS) project (10), were also derived to predict in-hospital mortality. However, these risk score models have some limitations (16–19). First, some of the excluded patients had a high risk, and some were modeled after selected populations that enrolled non-consecutive patients. Second, most risk scores were established in an era when the treatment strategy and patient characteristics were relatively different. Third, most of the published risk scores seldom contained patients from developing countries, especially in China.

Therefore, we aimed to develop and validate a novel in-hospital mortality risk model for patients with AMI from the Heart failure after Acute Myocardial Infarction with Optimal Treatment (HAMIOT) cohort in China. We also sought to build a simple risk score tool for in-hospital mortality that could be used prospectively for risk stratification.

## Materials and Methods

### Study Population

**FIGURE 1:**
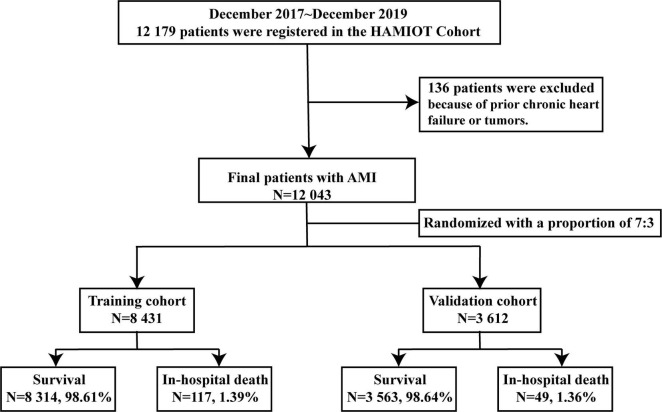
Study flow chart of the HAMIOT Cohort Study. From December 2017 to December 2019, 12,043 patients with AMI were randomly assigned into the training (*n* = 8,431) and validation (*n* = 3,612) cohorts. HAMIOT, the Heart failure after Acute Myocardial Infarction with Optimal Treatment; AMI, acute myocardial infarction.

With the support of National Key Research and Development Program of China, the HAMIOT cohort was a multicenter, prospective, observational cohort study that included consecutive patients with AMI in China^1^ (NCT03297164). From December 2017 to December 2019, a total of 12,179 patients aged 18 years or older with symptoms or signs of ST-segment elevation or non-ST-segment elevation were enrolled, in which 136 patients were excluded because of prior chronic heart failure or tumors. The cohort was randomly assigned into the training (*n* = 8,431) and validation (*n* = 3,612) cohorts. The proportion was 7:3. The overall study design and flow chart were presented in Figure 1. The study was evaluated in an external validation cohort (n = 3,095), with prospectively enrolled patients with AMI in the Second Affiliated Hospital of Harbin Medical University from October 2019 to March 2021.

In this study, variables such as demographic characteristics, medical history, presentation with electrocardiogram and echocardiography findings, laboratory examinations, and treatment strategies were collected during hospitalization. ST-segment elevation myocardial infarction (STEMI) and non-ST-segment elevation myocardial infarction (NSTEMI) were defined according to the ESC guidelines (11, 12). The endpoint was in-hospital all-cause mortality in patients with AMI. The data was collected and the patients were interviewed by a group of trained clinical research coordinators, cardiologists and nurses through an electronic data collection platform.

The study protocol was approved by the ethics or institutional review board of the hospitals participating in the study, and all procedures were in accordance with the Declaration of Helsinki. Each eligible patient signed an informed consent form and agreed to a follow-up after discharge either through over the telephone, inpatient, or outpatient interview.

### Statistical Methods

Generally, continuous variables were presented as median (25th and 75th percentiles), and were tested with the Student’s *t*-test or the Mann–Whitney *U* test. Categorical variables were expressed as counts and percentages (%), and were compared using the Chi-square(χ^2^) test or Fisher’s exact test. The training and validation cohorts were randomly divided using the method of Proc Surveyselect in SAS 9.3 (SAS Institute, Cary, NC, United States).

The unadjusted associations between candidate variables and in-hospital mortality were analyzed using the univariate logistic regression model. Variables, presented as *P* < 0.20 in the univariate logistic regression analysis, were included in the multivariate logistic regression analysis, and they were then evaluated by the stepwise selection approach for model building. The final logistic regression model contained variables with *P* values < 0.05. The associations between the candidate risk predictors and in-hospital mortality were presented as odds ratios (ORs) with 95% confidence intervals (CIs).

A novel simplified risk score was developed according to the final logistic regression model. For continuous variables, stratification was performed using certain thresholds, and the simplified risk score was re-evaluated. In the final logistic regression model, continuous variables were age, heart rate, systolic blood pressure (SBP), initial serum creatinine level and initial N-terminal pro-B-type natriuretic peptide (NT-proBNP) level. They were categorized as: (1) age (<60, 60–70, 70–80, ≥80 years); (2) heart rate (<60, 60–100, ≥100 beats/min), (3) SBP (<100, 100–120, 120–140, 140–160, ≥160 mmHg), (4) initial serum creatinine level (<1.3, ≥1.3 mg/dL), and (5) initial NT-proBNP level (<125, 125–2,000, >2,000 pg/mL). The risk score of each predictor was calculated on the basis of the beta (β) coefficient of the re-evaluated model (20).

Discrimination and calibration were evaluated using the c-statistic or receiver operating characteristic (ROC) curve and the Hosmer–Lemeshow goodness-of-fit test, respectively. Internal validation was evaluated using the bootstrap techniques (1,000 replications) to obtain optimism corrected c-statistics (21). External validation of the HAMIOT risk score was assessed using a prospectively subsequent AMI cohort from the Second Affiliated Hospital of Harbin Medical university. In addition, the performance of the HAMIOT risk score was assessed in selected subgroups, including age (<60 vs. ≥60 years), sex (female vs. male), body mass index (BMI) (<25 vs. ≥25 kg/m^2^), presentation with ST-segment elevation (STEMI vs. NSTEMI), current smoking (yes vs. no), history of stroke (yes vs. no), Killip class (I vs. II–IV), cardiac arrest (yes vs. no), and PCI treatment during hospitalization (yes vs. no). Moreover, the novel HAMIOT risk score was compared to the GRACE risk score in the training, validation and subgroup cohorts. Finally, we calculated the net reclassification improvement (NRI), which focused on the improvement that patients were appropriately assigned to different risk groups (low risk, score ≤ 50; intermediate risk, 50 < score ≤ 74; high risk, score > 74), and the integrated discrimination index (IDI), which evaluated how well the HAMIOT risk score increased prognostic accuracy (22).

The initial NT-proBNP level and cardiac enzymes were normalized using log_10_ transformation. The missing values of some prognostic variables were simply arranged according to the corresponding median or mode values. All statistical analysis were performed using the SAS 9.3 or R (version 4.1.0) software. All reported *P*-values were based on two-sided tests, and statistical significance was set at *P* < 0.05.

## Results

### Baseline Characteristic

From December 2017 to December 2019, a total of 12,179 patients with AMI (STEMI and NSTEMI) participated in the HAMIOT cohort. Among them, 136 patients were excluded because of prior chronic heart failure or tumors. In total, our study consisted of 12,043 eligible patients with AMI. The in-hospital mortality rate of these patients was 166 (1.38%). The median age was 62 years, 73% were male, and 70% presented with ST-segment elevation. The patients were randomly divided into the training (*n* = 8,431) and validation (*n* = 3,612) cohorts with in-hospital mortalities of 117 (1.39%) and 49 (1.36%), respectively. Demographic characteristics, medical history, presentation characteristics, laboratory examination results, medication and PCI treatment during hospitalization were described in Table 1. There were no significant differences between the training and validation cohorts (each *P* > 0.05).

**TABLE 1:** Characteristics of baseline in the training and validation cohorts.

Variables	All population	Training cohort	Validation cohort
Number of patients	12,043	8,431	3,612
**Demographic characteristics**			
Age, years	62.33(53.84,69.58)	62.21(53.60,69.48)	62.59(54.28,69.76)
Sex			
Male	8,758(72.72%)	6,172(73.21%)	2,586(71.59%)
Female	3,285(27.28%)	2,259(26.79%)	1,026(28.41%)
BMI, kg/m^2^	24.74(22.83,27.04)	24.77(22.84,27.05)	24.54(22.74,27.04)
**Medical history**			
Current smoking	4,952(41.12%)	3,482(41.30%)	1,470(40.70%)
History of diabetes	2,890(24.00%)	2,091(24.80%)	799(22.12%)
History of hypertension	6,167(51.21%)	4,340(51.48%)	1,827(50.58%)
History of CABG	51(0.42%)	40(0.47%)	11(0.30%)
History of PCI	781(6.49%)	542(6.43%)	239(6.62%)
History of stroke	1,634(13.57%)	1,109(13.15%)	525(14.53%)
**Presentation characteristics**			
**Presentation with STEMI**			
STEMI	8,434(70.03%)	5,913(70.13%)	2,521(69.80%)
NSTEMI	3,609(29.97%)	2,518(29.87%)	1,091(30.20%)
SBP, mmHg	130(116.00,148.00)	130(116.00,148.00)	130(116.00,149.00)
DBP, mmHg	80(70.00,90.00)	80(70.00,90.00)	80(70.00,90.00)
Heart rate, beats/min	75(65.00,86.00)	75(65.00,86.00)	75(65.00,87.00)
LVEF, %	58(50.00,62.00)	58(50.00,62.00)	58(50.00,62.00)
Cardiac arrest	105(0.87%)	76(0.9%)	29 (0.8%)
**Killip class**			
I	8,468(92.92%)	5,950(93.13%)	2,518(92.44%)
II–IV	645(7.08%)	439(6.87%)	206(7.56%)
**Laboratory examinations**			
White blood cell, 10^9^/L	9.8(7.82,12.20)	9.77(7.81,12.20)	9.81(7.84,12.19)
Red blood cell, 10^9^/L	4.62(4.24,5.00)	4.63(4.24,5.01)	4.6(4.23,4.98)
Hemoglobin, g/L	144(131.00,156.00)	144(131.00,156.00)	143(131.00,156.00)
Urea, mmol/L	5.60(4.54,6.90)	5.58(4.50,6.93)	5.64(4.60,6.90)
Serum creatinine, mg/dL	0.86(0.74,1.03)	0.86(0.74,1.03)	0.87(0.74,1.03)
ALT, U/L	27(18.00,42.00)	27(18.00,42.70)	26(17.00,42.00)
AST, U/L	47(25.50,117.00)	47(25.40,119.00)	47(25.80,113.20)
TG, mmol/L	1.46(1.03,2.11)	1.47(1.04,2.11)	1.45(1.01,2.09)
TC, mmol/L	4.53(3.86,5.26)	4.53(3.86,5.26)	4.52(3.88,5.27)
HDL-C, mmol/L	1.10(0.92,1.35)	1.10(0.92,1.34)	1.11(0.93,1.37)
LDL-C, mmol/L	2.72(2.09,3.39)	2.72(2.09,3.40)	2.71(2.09,3.38)
Fasting blood glucose, mg/dL	6.36(5.28,8.39)	6.38(5.29,8.44)	6.30(5.25,8.29)
NT-proBNP, pg/mL	602.15(180.00,1611.00)	606(182.00,1609.00)	598.6(173.00,1636.00)
CK, U/L	251(108.00,773.80)	248.5(107.00,778.00)	254(110.00,764.00)
CKMB, ng/mL	10.10(2.50,51.60)	10.20(2.50,51.60)	10.00(2.50,51.51)
cTn I, μg/L	1.98(0.28,11.60)	1.95(0.28,11.14)	2.05(0.30,12.60)
**Treatment during hospitalization**			
Aspirin	11,151(92.59%)	7,795(92.46%)	3,356(92.91%)
Clopidogrel/Ticagrelor	11,206(93.05%)	7,834(92.92%)	3,372(93.36%)
Statins	11,120(92.34%)	7,761(92.05%)	3,359(93.00%)
Absence of PCI treatment	3,356(27.87%)	2,337(27.72%)	1,019(28.21%)
**Primary endpoint**			
In-hospital mortality	166(1.38%)	117(1.39%)	49(1.36%)

*Continuous variables were presented as median (Q1, Q3 quantiles), and categorical variables were presented as number (%). BMI, body mass index; CABG, coronary artery bypass graft; PCI, percutaneous coronary intervention; STEMI, ST-segment elevation myocardial infarction; NSTEMI, non-ST-segment elevation myocardial infarction; SBP, systolic blood pressure; DBP, diastolic blood pressure; LVEF, left ventricular ejection fraction; ALT, alanine transaminase; AST, aspartate aminotransferase; TG, triglyceride; TC, total cholesterol; HDL-C, high-density lipoprotein cholesterol; LDL-C, low-density lipoprotein cholesterol; NT-proBNP, N-terminal pro-B-type natriuretic peptide; CK, creatine kinase; CK-MB, creatine kinase-MB; cTn I, cardiac troponin I.*

The external validation cohort contained 3,095 independent patients with AMI from the Second Affiliated Hospital of Harbin Medical University. Among them, 32 (1.03%) patients died in the hospital. Baseline characteristics were provided in Supplementary Material (Supplementary Table 1).

### Predictors of In-Hospital Mortality

In the training cohort, the association between each baseline characteristic and in-hospital mortality was analyzed using the univariate logistic regression model and was presented in Table 2 (for continuous variables) and Table 3 (for categorical variables). Patients who died in the hospital were more likely to be old, female, and had an elevated heart rate, low SBP and diastolic blood pressure, high incidence of previous diseases (diabetes, hypertension, coronary artery bypass graft, PCI treatment, and stroke), low rate of treatment with medication (aspirin, clopidogrel or ticagrelor, and statins) and PCI treatment (each *P* < 0.05). For laboratory findings, alanine transaminase, aspartate aminotransferase, and fasting plasma glucose were higher, and triglyceride, total cholesterol, and high-density lipoprotein cholesterol were lower in dead patients compared to survival patients (each *P* < 0.05). The initial levels of serum creatinine, NT-proBNP and cardiac enzymes (creatine kinase, creatine kinase-MB and cardiac troponin I) were high in the non-survival patients (each *P* < 0.05).

**TABLE 2:** Univariate analysis between baseline characteristics (continuous variables) and in-hospital mortality in the training cohort.

Variables	Patients alive	Patients died	OR (95%CI)	*P*-value
Number of patients	8,314	117	–	–
Age, years	62.06(53.50,69.30)	73.63(66.03,80.72)	1.11(1.08,1.13)	<0.01
BMI, kg/m^2^	24.79(22.86,27.06)	23.55(21.88,26.12)	0.93(0.88,0.98)	0.01
SBP, mmHg	130(116.00,148.00)	122(106.00,140.00)	0.98(0.98,0.99)	<0.01
DBP, mmHg	80(70.00,90.00)	77(65.50,85.00)	0.98(0.97,0.99)	<0.01
Heart rate, beats/min	75(65.00,86.00)	83(68.00,98.00)	1.02(1.01,1.03)	<0.01
LVEF,%	58(50.00,62.00)	46.4(40.00,57.00)	0.95(0.93,0.96)	<0.01
WBC, 10^9^/L	9.76(7.81,12.18)	10.35(7.90,14.15)	1.06(1.02,1.1)	<0.01
RBC, 10^9^/L	4.64(4.25,5.01)	4.29(3.92,4.64)	0.4(0.3,0.53)	<0.01
Hemoglobin, g/L	145(131.50,157.00)	133(123.00,148.00)	0.99(0.98,1)	0.02
Urea, mmol/L	5.57(4.50,6.90)	7.4(5.33,9.12)	1.00(1.00,1.01)	0.23
Serum creatinine, mg/dL	0.86(0.74,1.02)	0.96(0.76,1.36)	1.83(1.53,2.18)	<0.01
ALT, U/L	27(18.00,42.00)	37.65(21.00,61.00)	1.03(1.02, 1.04)^‡^	<0.01
AST, U/L	46.3(25.00,117.00)	86(37.00,222.30)	1.02(1.01, 1.03)^‡^	<0.01
TG, mmol/L	1.47(1.04,2.12)	1.35(1.02,1.84)	0.72(0.56,0.94)	0.01
TC, mmol/L	4.54(3.86,5.26)	4.23(3.41,4.89)	0.79(0.65,0.96)	0.02
HDL-C, mmol/L	1.10(0.92,1.34)	1.07(0.85,1.37)	0.55(0.32,0.95)	0.03
LDL-C, mmol/L	2.72(2.09,3.40)	2.54(1.92,3.26)	0.87(0.70,1.07)	0.17
Fasting blood glucose, mg/dL	6.37(5.29,8.42)	7.49(6.10,11.04)	1.09(1.05,1.14)	<0.01
NT-proBNP, pg/mL	592(180.00,1564.93)	3600(1377.00,9080.00)	2.16(1.87,2.49)*	<0.01
CK, U/L	245(107.00,769.00)	508(186.00,1725.00)	2.28(1.59,3.27)*	<0.01
CKMB, ng/mL	10(2.50,50.90)	29(8.20,181.80)	2.06(1.42, 2.97)*	<0.01
cTn I, μg/L	1.9(0.27,11.00)	5.77(1.08,29.53)	1.44(1.17,1.77)*	<0.01

*^‡^Odds ratio of per-10 unit increase with 95% confidence interval.*

*Odds ratio of log_10_ transformation with 95% confidence interval.

*OR, odds ratio; other abbreviations are in Table 1.*

**TABLE 3:** Univariate analysis between baseline characteristics (categorical variables) and in-hospital mortality in the training cohort.

Variables		n	In-hospital mortality,%	OR (95%CI)	*P*-value
**Demographic characteristics**					
Sex	Male	6172	0.97	Ref	Ref
	Female	2259	2.52	2.64(1.83, 3.80)	<0.01
**Medical history**					
Current smoking	No	4949	1.72	Ref	Ref
	Yes	3482	0.92	0.53(0.35,0.8)	0.02
History of diabetes	No	6340	1.21	Ref	Ref
	Yes	2091	1.91	1.59(1.08,2.33)	0.02
History of hypertension	No	4091	1.08	Ref	Ref
	Yes	4340	1.68	1.57(1.08,2.29)	0.02
History of CABG	No	8391	1.37	Ref	Ref
	Yes	40	5	3.79(0.9,15.89)	0.07
History of PCI	No	7889	1.24	Ref	Ref
	Yes	542	3.51	2.89(1.75,4.76)	<0.01
History of stroke	No	7322	1.22	Ref	Ref
	Yes	1109	2.52	2.11(1.37,3.23)	0.01
**Presentation characteristics**					
Presentation with STEMI	NSTEMI	2518	1.11	Ref	Ref
	STEMI	5913	1.51	1.36(0.89, 2.08)	0.16
Cardiac arrest	No	8355	1.38	Ref	Ref
	Yes	76	2.63	0.94(0.47,7.99)	0.36
Killip class	I	5950	1.46	Ref	Ref
	II–IV	439	4.33	3.05(1.84,5.06)	<0.01
**Treatment during hospitalization**					
Aspirin	No	636	3.3	Ref	Rref
	Yes	7795	1.23	0.37(0.23,0.59)	<0.01
Clopidogrel/Ticagrelor	No	597	3.18	Ref	Ref
	Yes	7834	1.25	0.39(0.23,0.63)	<0.01
Statins	No	670	2.84	Ref	Ref
	Yes	7761	1.26	0.44(0.27,0.72)	0.01
Absence of PCI treatment	No	6094	0.64	Ref	Ref
	Yes	2337	3.34	5.36(3.64,7.90)	<0.01

*Abbreviations are in Tables 1, 2.*

**FIGURE 2:**
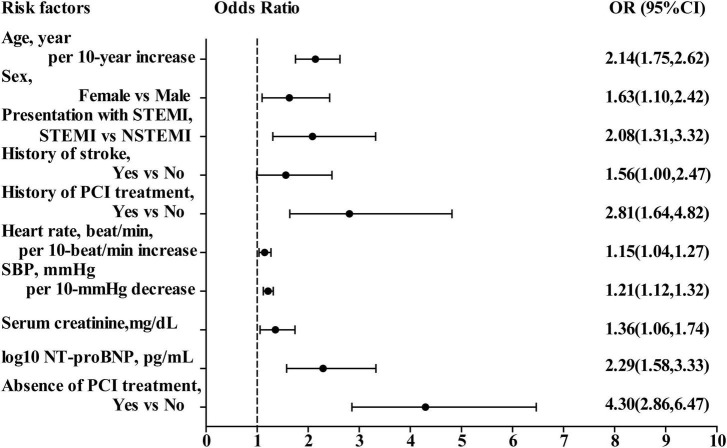
Odds ratio of in-hospital mortality in multivariate logistic regression model. OR, odds ratio; CI, confidence interval; STEMI, ST-segment elevation myocardial infarction; NSTEMI, non-ST-segment elevation myocardial infarction; SBP, systolic blood pressure; NT-proBNP, N-terminal pro-B-type natriuretic peptide; PCI, percutaneous coronary intervention.

In the multivariate logistic regression analysis, variables presented as *P* < 0.20 in the univariate analysis (Tables 2, 3) were included. Ten predictors were found to be independently associated with in-hospital mortality: age, sex, history of PCI treatment, history of stroke, presentation with ST-segment elevation, heart rate, SBP, initial serum creatinine level, initial NT-proBNP level, and PCI treatment. The results of the multivariate logistic regression analyses were displayed in Figure 2. The performance of discrimination and calibration were 0.88 (c-statistic) and *P* = 0.16 (Hosmer–Lemeshow goodness-of-fit test), respectively.

### HAMIOT Risk Score

**FIGURE 3:**
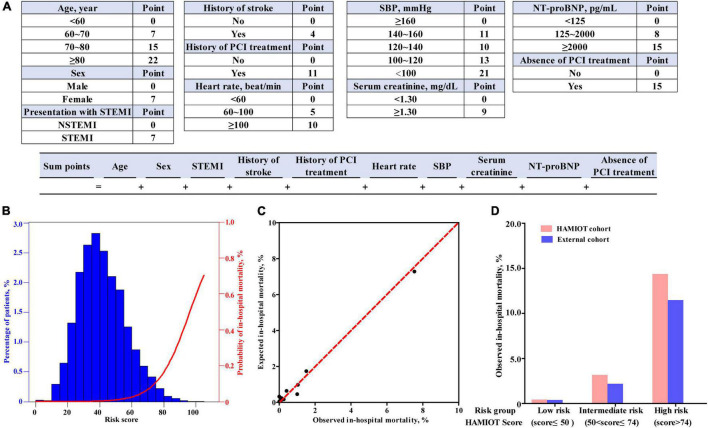
**(A)** Risk score calculator of in-hospital mortality for patients with AMI. **(B)** Distribution of HAMIOT risk score and the probability of in-hospital mortality. **(C)** Relationship between the observed and expected in-hospital mortality across deciles of risk. **(D)** Observed in-hospital mortality of HAMIOT cohort and external cohort stratified by three risk groups (low risk, intermediate risk, and high risk groups). The observed in-hospital mortality rates were 0.35, 3.09, and 14.29% in the HAMIOT cohort, and 0.31, 2.21, and 11.39% in the external cohort, respectively. HAMIOT, the Heart failure after Acute Myocardial Infarction with Optimal Treatment, STEMI, ST-segment elevation myocardial infarction; NSTEMI, non-ST-segment elevation myocardial infarction; SBP, systolic blood pressure; NT-proBNP, N-terminal pro-B-type natriuretic peptide; PCI, percutaneous coronary intervention.

The novel HAMIOT risk score compromised predictors identified in the multivariate logistic regression model and was re-evaluated by categorical predictors, including age, heart rate, SBP, initial serum creatinine level and initial NT-proBNP level. The score of each predictor was built on the basis of estimated β coefficient parameter. The results with simple inter score were shown in Figure 3A. The c-statistic of the simplified HAMIOT risk score was 0.88. Figure 3B showed the distribution of individual scores along with the relationship between the patient risk score and the probability of in-hospital mortality in the training cohort. Figure 3C presented the corresponding relationship between the observed and expected in-hospital mortality across deciles of risk, in which the Hosmer–Lemeshow goodness-of-fit test was *P* = 0.35.

Discrimination of the HAMIOT risk score was validated both internally and externally. The new simplified risk score was validated in the validation cohort (*n* = 3,612), and the discrimination ability was 0.82 with good calibration (*P* = 0.64). Internal validation was also evaluated using bootstrap techniques (1,000 replications) to obtain the optimism corrected c-statistic, which was 0.85. The discrimination ability was 0.82 (c-statistic) in the external validation cohort, and the ROC curve was presented in Supplementary Figure 1. In addition, the c-statistic values of the selected subgroups were calculated, and they performed well, as seen in Table 4.

**TABLE 4:** The performance of discrimination ability in the subgroups between the HAMIOT risk score and GRACE risk score.

Subgroups	Training cohort			Validation cohort			All patients		
			
	n	GRACE	HAMIOT	n	GRACE	HAMIOT	n	GRACE	HAMIOT
Age, years									
∼60	3600	0.88	0.84	1485	0.57	0.81	5085	0.8	0.84
60∼	4831	0.72	0.85*	2127	0.65	0.77*	6958	0.7	0.83*
Sex									
Male	2259	0.7	0.83*	1026	0.68	0.75	3285	0.7	0.81*
Female	6172	0.86	0.89	2586	0.72	0.85*	8758	0.81	0.88*
**Presentation with STEMI**									
STEMI	5913	0.8	0.88*	2521	0.73	0.81*	8434	0.78	0.86*
NSTEMI	2518	0.83	0.87	1091	0.72	0.81	3609	0.81	0.85
BMI, kg/m^2^									
<25	4554	0.76	0.86*	2024	0.72	0.83*	6578	0.75	0.85*
≥25	3877	0.86	0.89	1588	0.71	0.79	5465	0.82	0.86
**Current smoking**									
No	4949	0.77	0.85*	2142	0.72	0.78	7091	0.75	0.83*
Yes	3482	0.86	0.92	1470	0.66	0.89*	4952	0.82	0.91*
**History of stroke**									
No	7322	0.81	0.88*	3087	0.72	0.82*	10409	0.79	0.87*
Yes	1109	0.76	0.81	525	0.7	0.76	1634	0.74	0.8
**Killip class**									
I	5950	0.79	0.88*	2518	0.65	0.77*	8468	0.75	0.85*
II–IV	439	0.75	0.79	206	0.82	0.79	645	0.78	0.79
Cardiac arrest									
No	8355	0.81	0.87*	3583	0.72	0.82*	11938	0.78	0.86*
Yes	76	0.97	0.98	29	0.84	0.89	105	0.94	0.96
**PCI treatment during hospitalization**									
No	2337	0.8	0.84	1019	0.72	0.78	3356	0.78	0.82*
Yes	6094	0.78	0.83	2593	0.71	0.79	8687	0.76	0.82*

Abbreviations are in Tables 1, 2. *P < 0.05 between the HAMIOT risk score vs the GRACE risk score.

Furthermore, the total risk score of the established HAMIOT risk score model ranged from 0 to 121. The novel HAMIOT risk score of in-hospital mortality was stratified into three risk groups: low risk(score ≤ 50); intermediate risk(50<score ≤ 74); and high risk(score > 74). Figure 3D described the observed in-hospital mortality across each risk group in the HAMIOT and external cohorts. The observed in-hospital mortality rates were 0.35, 3.09, and 14.29% in the HAMIOT cohort, and 0.31, 2.21, and 11.39% in the external cohort, respectively.

### Comparision With Grace Risk Score

**FIGURE 4:**
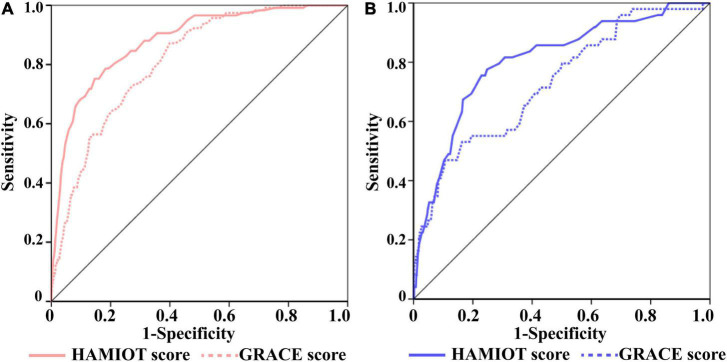
The ROC Curves of the HAMIOT and the GRACE risk score. **(A)** In the training cohort, the c-statistic in the HAMIOT score 0.88(0.84,0.91) was higher than the GRACE score 0.81(0.77,0.84). **(B)** In the validation cohort, the c-statistic in the HAMIOT score 0.82(0.76,0.88) was higher than the GRACE 0.72(0.65,0.8). HAMIOT, the Heart failure after Acute Myocardial Infarction with Optimal Treatment; GRACE, the Global Registry of Acute Coronary Events; ROC, receiver operating characteristic.

Compared with the GRACE risk score, the c-statistic of the HAMIOT risk score was 0.88 vs. 0.81 (Figure 4A) in the training cohort and 0.82 vs. 0.72 (Figure 4B) in the validation cohort, presenting a significantly improved discrimination ability. The improved reclassification and discrimination were evaluated using NRI and IDI, and they were 30.81 and 4.9% (each *P* < 0.01), respectively. For subgroups, such as age (≥60 years), presentation with STEMI, BMI (<25 kg/m^2^), absence of previous stroke, Killip class(I level), and presentation without cardiac arrest, the c-statistic values were higher than the GRACE risk score (each *P* < 0.05). The other subgroups presented comparable discrimination ability with the GRACE risk score.

## Discussion

The present study extended clinical understanding of AMI risk and provided a novel simplified HAMIOT risk score of in-hospital mortality of 12,043 consecutive patients with AMI in China. The main findings were as follows: (1) Ten independent predictors were found in the final logistic regression model that include age, sex, history of PCI treatment, history of stroke, presentation with STEMI, heart rate, SBP, initial serum creatinine level, initial NT-proBNP level and PCI treatment during hospitalization; (2) The novel HAMIOT risk score was established to predict in-hospital mortality and showed excellent discrimination and calibration ability; (3) Similar discrimination capacity was found in the validation cohorts (internal and external), and in various important clinical subgroups, such as age, sex, BMI, presentation with STEMI, current smoking, history of stroke, Killip class, cardiac arrest, and PCI treatment during hospitalization; (4) The new simplified risk score model improved discrimination ability compared with the GRACE score and provided a clinically convenient risk stratification tool for future patients with AMI.

Several risk score models have been developed to predict in-hospital mortality and have presented excellent performance of risk stratification in patients with ACS/AMI. Among them, the GRACE risk score (2) has been extensively recommended and used in clinical practice. The GRACE risk score was derived from an international registry of non-consecutive patients with ACS from 1999 to 2001, and the in-hospital mortality was 4.6%, which was higher than the mortality of 1.6% in the HAMIOT cohort. There were several reasons for the low mortality rates in this study. First, the GRACE registry was performed nearly 20 years earlier, and the treatment strategy, such as PCI treatment, was relatively less frequently used (less than 30%) (23) than in the HAMIOT cohort (72.13%). Second, the risk factors of the population with AMI included in the HAMIOT cohort have changed over time. Compared with the GRACE cohort, patients in our study were younger, had fewer females and smokers, and had higher incidence of previous diseases (diabetes, hypertension, PCI, and coronary artery bypass graft). Third, the contributing hospitals participating in the HAMIOT cohort tended to be chest pain centers. Fan et al. (24) and Sun et al. (25) reported that chest pain center accreditation presented a higher PCI treatment rate and a low short term all-cause mortality in patients with AMI in China. Thus, with the low mortality rate and different risk factors, an updated risk score is necessary for the current clinical practice.

The HAMIOT risk score contained 10 independent predictors, including age, sex, history of PCI treatment, history of stroke, presentation with STEMI, heart rate, SBP, initial serum creatinine level, initial NT-proBNP level and PCI treatment). Among the variables, age, heart rate, serum creatinine, and SBP were mainly confirmed in some risk scores (2, 4, 6, 8). There were initially six new predictors included in the HAMIOT risk score: sex, history of PCI treatment or stroke, presentation with STEMI, initial NT-proBNP level and absence of PCI treatment during hospitalization.

For sex, female patients with AMI had been demonstrated to have a higher risk of short-term and long-term mortality than male patients in previous studies (26–30); however, the adjusted risk between sex and mortality was not clear. In our study, females had a higher in-hospital mortality risk than males (2.52 vs. 0.97%), and the OR was 1.63(1.10, 2.42) after adjustment for other predictors. This means that the risk of death was increased by 63% in female patients compared to male patients. In our study, we found that patients with previous diseases (diabetes, hypertension, coronary artery bypass graft, PCI or stroke), presented with high in-hospital mortality. Among them, patients with previous PCI treatment or stroke had an increased risk of death by 81 and 56%, respectively. For patients with STEMI, risk was not found for the presence of ST-segment elevation at the time of presentation in the GRACE risk score (2). However, the ACTION Registry–GWTG mortality risk model (6) reported that patients with STEMI had an approximately 80% higher risk than patients with NSTEMI. Considering the inconsistency of risks, patients with ST-segment elevation were considered in our study. In the univariate analysis, in-hospital mortality rates of patients with STEMI and NSTEMI were similar (1.51 and 1.11%, respectively). However, the association between STEMI and mortality was 2.08 (1.31, 3.32) in the multivariate analysis, presenting a 2.08-fold increased risk. The increased initial NT-proBNP level had proven to be an important predictor of early and late mortality (31), and has been recommended as a prognostic indication of death and heart failure (14, 32). The association between log_10_(NT-proBNP) and in-hospital mortality in our study was presented as 2.16 (1.87, 2.49) in the univariate analysis and 2.29 (1.58, 3.33) in the multivariate analysis. PCI has been widely proven and recommended for patients with AMI (13, 14), and some risk models of long-term mortality have been established and validated for patients with ACS undergoing PCI treatment (33, 34). While PCI treatment has an obvious benefit for patient with AMI, risk models rarely considered it a predictor of in-hospital mortality. Moreover, the proportion of PCI treatment was relatively low (50%) for patients with AMI in China (25), which could lead to serious outcomes, such as cardiac arrest or even death in hospital. In our study, the absence of PCI treatment was included, and the in-hospital mortality between the absence and presence of the PCI treatment were 3.34 and 0.64%, respectively. The adjusted OR was 4.30 (2.86, 6.47), showing a 4.3-fold increased risk of death. Other factors known to be associated with in-hospital mortality, including Killip class and cardiac arrest (2–4, 8, 11), were considered in the logistic regression model, but hardly contributed to in-hospital mortality.

The simplified HAMIOT risk score presented excellent discrimination and calibration, and it was better than the GRACE risk score in the training and validation (internal and external) cohorts. The c-statistic was comparable to other risk scores in patients with AMI (2, 4, 6, 10). In our study, the HAMIOT risk score had higher c-statistic values compared with the Simplified CAMI-NSTEMI and CCC-ACS scores which were built based on Chinese patients with AMI in the HAMIOT cohort (training cohort: 0.88 vs. 0.72, 0.88 vs. 0.82; validation cohort: 0.82 vs. 0.74, 0.82 vs. 0.78). Moreover, the simplified risk score performed well in several subgroups, especially in patients with smoking or cardiac arrest (the Harrel’s c-statistics were higher than 0.90). Besides, the c-statistic in the novel risk score was better than the GRACE risk score, especially in some subgroups (older, presentation with STEMI, normal BMI index, absence of previous stroke, I level of Killip class and presentation without cardiac arrest). Thus, the novel HAMIOT risk score may be more useful to predict in-hospital mortality.

### Study Limitations

Although the HAMIOT risk score is a novel and practical tool that can stratify the risk of in-hospital mortality in patients with AMI, it has several limitations. First, the HAMIOT score was based on Chinese population, whether it can be applied to other ethnicities needs further validation. Second, although we validated our risk score model in the validation and an independent prospective external cohort, the HAMIOT risk score needs to be verified in large cohorts. Third, patients with a history of chronic heart failure were excluded from our study, clinicians should take special caution when applying these results to these patients. Fourth, since the HAMIOT risk score only assessed in-hospital mortality, long-term mortality risk predictors should be further studied.

## Conclusion

In conclusion, the HAMIOT risk score demonstrated that the risk of in-hospital mortality in patients with AMI could be reliably predicted using 10 highly predictive variables. All of these variables could be easily obtained during hospitalization. Since the novel risk score tool is simple and easy to calculate, clinicians can rapidly apply to predict the risk of mortality and to provide the correct therapies or management strategies.

## Data Availability Statement

The original contributions presented in the study are included in the article/Supplementary Material, further inquiries can be directed to the corresponding authors.

## Ethics Statement

The studies involving human participants were reviewed and approved by the Ethics Committee of the Second Affiliated Hospital of Harbin Medical University. The patients/participants provided their written informed consent to participate in this study.

## Author Contributions

LlL contributed to the conception, design and statistical analyses of the study. XZ, YW, XY, CL, WZ, WY, BL, LxL, and NT screened for eligible patients and conducted the data acquisition of the cohort. HJ and JH contributed to the critical manuscript revision. BY and KL were substantial contribution to the design of research and critical manuscript revision. All authors contributed to manuscript revision, and they have read and approved the submitted version.

## Conflict of Interest

The authors declare that the research was conducted in the absence of any commercial or financial relationships that could be construed as a potential conflict of interest.

## Publisher’s Note

All claims expressed in this article are solely those of the authors and do not necessarily represent those of their affiliated organizations, or those of the publisher, the editors and the reviewers. Any product that may be evaluated in this article, or claim that may be made by its manufacturer, is not guaranteed or endorsed by the publisher.

## Footnotes

1
https://clinicaltrials.gov
