# Supplementary material for: A Novel Risk Score to Predict In-Hospital Mortality in Patients With Acute Myocardial Infarction: Results From a Prospective Observational Cohort
Source: Front Cardiovasc Med. 2022 Apr 7;9:840485. doi: 10.3389/fcvm.2022.840485 (PMC9021415; doi:10.3389/fcvm.2022.840485)
Supplement: Supplementary file 1 [file Data_Sheet_1.pdf]

Supplementary Material

1 Supplementary Figures and Tables

1.1 Supplementary Figures

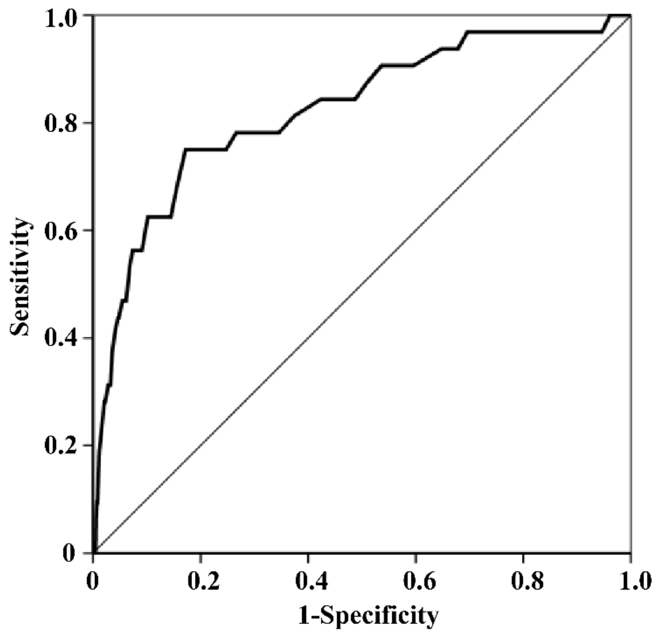

Supplementary Figure 1. The ROC Curve of the HAMIOT risk score in the external cohort. The Harrell's c-index was 0.82(0.74,0.91). Abbreviations: HAMIOT=the Heart failure after Acute Myocardial Infarction with Optimal Treatment; ROC=Receiver operating characteristic.

1.2 Supplementary Tables

Supplementary Table 1 Baseline characteristics of the external cohort.

Variables	External cohort	Patients alive	Patients died	*P*
No. of patients	3095	3063	32	
Demographic characteristics				
Age, years	62.08(53.65,68.98)	62.03(53.58,68.92)	68.69(65.08,74.23)	<0.01
Sex				
Male	2196(70.95%)	2178(71.11%)	18(56.25%)	0.07
Female	899(29.05%)	885(28.89%)	14(43.75%)	
BMI, Kg/m^2	24.77(22.57,27.45)	24.77(22.58,27.47)	22.86(21.36,25.95)	0.03
Medical history				
Current smoking	1419(45.85%)	1413(46.13%)	6(18.75%)	<0.01

History of diabetes	792(25.59%)	785(25.63%)	7(21.88%)	0.63
History of hypertension	1610(52.02%)	1589(51.88%)	21(65.63%)	0.12
History of CABG	8(0.26%)	8(0.26%)	0(0.00%)	1.00
History of PCI	242(7.82%)	241(7.87%)	1(3.13%)	0.32
History of stroke	603(19.48%)	588(19.20%)	15(46.88%)	<0.01
Presentation characteristics				
Presentation with STEMI				
STEMI	1885(60.90%)	1858(60.66%)	27(84.38%)	0.01
NSTEMI	1210(39.10%)	1205(39.34%)	5(15.63%)	
SBP, mmHg	132(118.00,150.00)	132(118.00,150.00)	120.5(100.50,137.00)	<0.01
DBP, mmHg	83(72.00,90.00)	83(72.00,90.00)	77(67.50,88.00)	0.02
Heart rate, beats/min	80(68.00,87.00)	80(68.00,86.00)	91(84.50,100.00)	<0.01
LVEF, %	59(51.00,62.00)	59(51.00,62.00)	46(39.00,51.80)	<0.01
Cardiac arrest	16(0.52%)	2(0.07%)	14(43.75%)	<0.01
Laboratory examination				
White blood cell, 10^9/L	10.3(8.20,12.90)	10.3(8.10,12.80)	14.25(10.90,17.50)	<0.01
Red blood cell, 10^9/L	4.54(4.18,4.92)	4.54(4.18,4.92)	4.24(3.62,4.84)	0.01
Hemoglobin, g/L	143(130.00,155.00)	143(130.00,155.00)	129(113.00,150.50)	<0.01
Serum creatinine,	0.9(0.77,1.10)	0.9(0.77,1.09)	1.28(1.02,1.68)	<0.01
NT-proBNP, pg/mL	573(170.00,1935.00)	565.5(169.00,1912.50)	4072.5(1045.50,7941.50)	<0.01
PCI treatment during hospitalization				
Absence of PCI	782(25.27%)	770(25.14%)	12(37.50%)	0.11

Continuous variables are presented as median (Q1, Q3 quantiles), and categorical variables are presented as number (%). BMI=Body mass index; CABG=coronary artery bypass graft; PCI=percutaneous coronary intervention; STEMI=ST-segment elevation myocardial infarction; NSTEMI=non-ST-segment elevation myocardial infarction; SBP=Systolic blood pressure; DBP= Diastolic blood pressure; LVEF= Left ventricular ejection fraction; NT-proBNP=N- terminal pro-B-type natriuretic peptide.
